# On the Use of the Nitrate of Silver in Lacerated Wound of the Face

**Published:** 1851-10

**Authors:** 


### ARTICLE XXI.

### *On the Use of the Nitrate of Silver in Lacerated Wound of the Face.*

Miss R.——, aged about twenty. On a very windy day, a piece of slate was blown from the roof of a house, which fell upon her forehead, and inflicted a wound of five inches in length, commencing on the forehead, above the right eye, passing obliquely across the nose, and terminating on the left cheek, leaving a large open wound, quite disfiguring the face. The wounded parts after being well cleansed from extraneous matter, were neatly closed by the interrupted suture; the nitrate of silver was then applied along the edges of the wound, on the line of the wound, and also on the surrounding skin. Afterwards, strips of adhesive plaster were applied, without any other covering.

The wound healed by the first intention, and required no further application. It is now several years since the accident. A common observer, standing at a short distance, cannot see the mark of the union, for no disagreeable mark or cicatrix remains.

Great advantages are derived from healing lacerated wounds on the face by the aid of the nitrate of silver.

1. It prevents irritation arising from the irregular edges of a lacerated wound, and it induces adhesive inflammation as readily as in an incised wound.

2. The inflammation, swelling and irritative fever, consequent on lacerated wounds, are in a great measure prevented, and there being no ulcerative process, there is no loss of substance, so that unsightly scars and raised cicatrices are obviated.—*London Lancet.*

